# Environmental Change Is Reshaping the Temperature Sensitivity of Sesquiterpene Emissions and Their Atmospheric Impacts

**DOI:** 10.1111/gcb.70258

**Published:** 2025-06-04

**Authors:** Efstratios Bourtsoukidis, Alex Guenther, Hui Wang, Theo Economou, Georgia Lazoglou, Aliki Christodoulou, Theo Christoudias, Anke Nölscher, Ana M. Yañez‐Serrano, Josep Peñuelas

**Affiliations:** ^1^ Climate and Atmosphere Research Center (CARE‐C) The Cyprus Institute Nicosia Cyprus; ^2^ Department of Earth System Science University of California Irvine USA; ^3^ Department of Mathematics and Statistics University of Exeter Exeter UK; ^4^ Bayreuth Center of Ecology and Environmental Research University of Bayreuth Bayereuth Germany; ^5^ Institute of Environmental Assessment and Water Research (IDAEA) Barcelona Spain; ^6^ Center for Research Ecology and Forestry Applications (CREAF) Barcelona Spain; ^7^ Global Ecology Unit CREAF‐CSIC‐UAB Barcelona Spain

**Keywords:** atmospheric chemistry, biogenic volatile organic compounds (BVOCs), biosphere‐atmosphere interactions, ecological interactions, environmental change, sesquiterpenes

## Abstract

Air temperature is a critical regulator of ecosystem functions, including the release of biogenic volatile organic compounds (BVOCs) that mediate biosphere‐atmosphere interactions. Among these, sesquiterpenes (SQTs) stand out for their dual role as ecologically significant compounds and highly reactive atmospheric constituents. Despite the inherently complex relationship between temperature and biogenic emissions, global emission estimates rely on simplistic parameterizations, assuming a fixed exponential response across all ecosystems and environmental conditions. Here, we synthesize two decades (1997–2019) of SQT emission studies, uncovering significant variability in temperature responses and basal emission rates driven by plant functional types (PFTs) and diverse environmental co‐factors. When PFT‐dependent parameterizations are integrated into emission‐chemistry simulations, the results reveal sensitive feedbacks on atmospheric processes, including ground‐level ozone (O_3_) production and secondary organic aerosol (SOA) formation. Surprisingly, we identify a statistically significant decline in SQT temperature responses over time, suggesting that evolving environmental changes are reshaping the fundamental relationship between temperature and SQT emissions. This meta‐analysis highlights the temperature sensitivity of sesquiterpenes (β_SQT_) as a key parameter at the interface of the biosphere, abiotic and biotic environmental change, and atmospheric processes, with cascading effects on air quality and climate. Our findings emphasize the potential to consider β_SQT_ as a “volatile stressometer” for ecosystem‐atmosphere interactions, where environmental stresses regulate the emission responses, with cascading effects on atmospheric chemistry and wider implications for future climate‐vegetation feedbacks.

## Graphical abstract

Environmental change is altering how plants emit sesquiterpenes, a group of highly reactive biogenic volatile organic compounds (BVOCs) that influence both ecosystem function and atmospheric chemistry. This meta‐analysis shows that environmental drivers are altering how sesquiterpene emissions respond to temperature, with significant implications for atmospheric processes. As a result, the exchange of gases between vegetation and the atmosphere is shifting in ways that affect air quality, chemical reactions, and climate‐related feedbacks. These findings suggest that temperature sensitivity in these emissions may serve as a volatile stressometer for changing plant–atmosphere interactions.

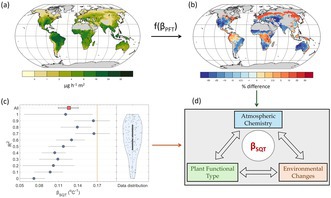

## 1 Introduction

Sesquiterpenes (SQTs; C_15_H_24_) are highly reactive biogenic volatile organic compounds (BVOCs) emitted primarily by global vegetation (Duhl et al. 2008), with additional contributions from soils (Bourtsoukidis et al. 2018) and cryptogamic organisms (Edtbauer et al. 2021). Despite their low atmospheric abundance at parts‐per‐trillion (ppt) levels, they play an outsized role in atmospheric chemistry. Their chemical reactivity and relatively large size for a BVOC make them effective precursors of secondary organic aerosols (SOA) and cloud condensation nuclei (CCN), which influence cloud formation, the Earth's radiative balance, and thereby impact both climate and air quality (Bonn 2003; Hallquist et al. 2009). SQTs contribute to the oxidative capacity of the atmosphere by reacting with hydroxyl (OH) and nitrate (NO_3_) radicals, producing low‐volatility oxidation products that drive SOA formation and growth (Barreira et al. 2021; Jaoui et al. 2013). They also interact with ground‐level ozone (O_3_), acting as both a source, through photochemical reactions with nitrogen oxides (NO_x_), and a sink via chemical degradation in leaf surfaces (Atkinson and Arey 2003; Bourtsoukidis et al. 2012; Fares et al. 2008; Ossola and Farmer 2024).

Plants emit SQTs under the influence of both biotic and abiotic factors, each playing a vital role in ecosystem dynamics. Biotically, SQTs are a key component of a plant's defense system, released in response to herbivory or pathogen attacks to deter predators or attract the natural enemies of herbivores (Gershenzon and Dudareva 2007; Hu et al. 2021; Sharma et al. 2017). They also play a crucial role in mediating plant–plant and plant‐pollinator interactions, thereby shaping ecosystems at multiple scales (Boncan et al. 2020; Kantsa et al. 2018; Schuman 2023). Abiotically, their release is influenced by environmental factors such as heat, drought, ozone, and mechanical damage, reflecting the plant's adaptive responses to changing conditions (Agathokleous et al. 2020; Bourtsoukidis 2014; Holopainen and Gershenzon 2010; Loreto and Schnitzler 2010; Niinemets et al. 2010). Among abiotic factors, temperature exerts the strongest control over SQT emissions by modulating their biosynthesis and enhancing volatilization rates, leading to higher fluxes (Copolovici and Niinemets 2015; Duan et al. 2021; Gershenzon and Dudareva 2007). As temperatures rise, these emissions intensify, amplifying their role in atmospheric chemistry and strengthening the connection between vegetation processes, air quality, and climate (Henrot et al. 2017; Peñuelas and Staudt 2010).

This critical influence on the Earth's system has driven the development of global emission models, such as the Model of Emissions of Gases and Aerosols from Nature (MEGAN) (Guenther et al. 2012), which incorporate temperature‐driven enzymatic activity and light‐dependent electron transport as primary mechanisms regulating emissions (Guenther et al. 2006). MEGAN offers a mechanistic yet computationally efficient framework for large‐scale simulations, with refinements to include additional environmental factors such as leaf age, soil moisture, and CO_2_ inhibition (Guenther et al. 2020). Nevertheless, temperature remains the dominant predictor of SQT emissions, encapsulated in the empirical temperature sensitivity parameter (β_SQT_), expressed as:

(1) $$E_{\mathrm{SQT}}=E_{s}\exp\left(\beta_{\mathrm{SQT}}\;\left(T-T_{s}\right)\right)$$

Here, Es denotes the emission potential at standard conditions (*s* = 30°C), while β_SQT_ is an empirical, constant coefficient (β_SQT_ = 0.17°C^−1^) that quantifies the temperature‐emission relationship, commonly referred to as temperature sensitivity. This meta‐analysis aims to examine β_SQT_ by synthesizing experimentally derived values from the literature, identifying key environmental factors that influence its spatial and temporal variability, and evaluating its broader implications for atmospheric chemistry. Ultimately, we seek to determine whether the temperature sensitivity of SQT emissions can provide insights into vegetation dynamics and their adaptive strategies under environmental change.

## 2 Temperature and Sesquiterpene Emissions

### 2.1 The Temperature‐Driven Variability in Sesquiterpene Emissions

**FIGURE 1:**
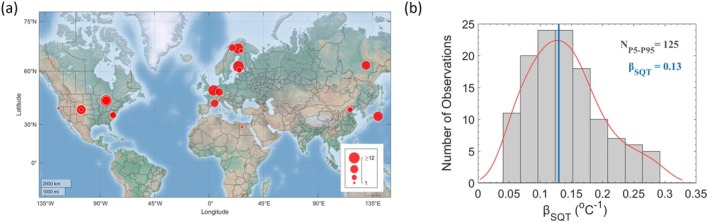
Spatial Observations Across Literature. (a) Map of experimentally derived temperature sensitivities for sesquiterpenes (β_SQT_), with the size of the red circles indicating the number of data points collected at each location. (b) Histogram of reported β_SQT_ values within the 5th to 95th percentiles. The red curve represents a kernel density estimate (KDE), with the peak indicating the central tendency of the β_SQT_ distribution. Map lines delineate study areas and do not necessarily depict accepted national boundaries.

A comprehensive review of SQT‐indexed publications in the Web of Science following the approach of Bourtsoukidis et al. (2024) spanning two decades (2001–2021) identified 139 β_SQT_ coefficients derived from 24 studies that performed regression analyses between temperature and emission responses across 19 diverse locations worldwide (Figure 1a). These coefficients exhibited substantial variability, ranging from 0.009 to 1.63 °C^−1^ (Figure S1), but no statistically significant differences were found between offline and online methods, or between enclosure‐based and ecosystem‐level measurements. This suggests that the compiled values can be reasonably compared in the context of our synthesis. To ensure robust statistical analyses, we excluded extreme outliers, retaining the ones that fall within the 5th to 95th percentiles, resulting in a total of 125 samples. This filtered dataset yielded a median β_SQT_ value of 0.13°C^−1^ ± 0.06°C^−1^, closely aligning with the central tendency observed in the kernel density estimate (KDE) distribution (Figure 1b).

All documented observations of β_SQT_ originate exclusively from the Northern Hemisphere, with the majority being derived from temperate (67%) and boreal (25%) ecosystems (Figure S2). The remaining data come from a single study on Arctic wetlands with mixed vegetation (Hellén et al. 2020). Although β_SQT_ did not differ between temperate and boreal ecosystems (Figure S3), Arctic wetlands exhibited significantly lower temperature responses (*p* < 0.001) compared to the rest of the data. Notably, no measurements have been reported from tropical forests, despite their dense vegetation, rich biodiversity, and ecological significance (Artaxo et al. 2022; Gibson et al. 2011; Yáñez‐Serrano et al. 2020), highlighting a critical geographical gap in the data.

The observational gaps extend to the seasonal coverage (Figure S4). Nearly half of all measurements (48%) were conducted in summer, when favorable weather conditions facilitate experimental observations, and emissions are expected to be higher. A quarter of the data spans multiple seasons, providing insights into how temperature responses change over seasons. Some studies have indicated that during spring recovery, SQT emissions increase while β_SQT_ decreases, suggesting that developmental priorities, rather than temperature alone, regulate emission responses (Fares et al. 2011; Hakola et al. 2006; Hellén et al. 2020; Matsunaga et al. 2011; Ruuskanen 2005; Tarvainen et al. 2005). This phenomenon has been clearly demonstrated for *Norway spruce*, where daily β_SQT_ values were considerably reduced during needle expansion (Bourtsoukidis et al. 2012). Notably, during the growing period, the composition of speciated SQTs is also changing (Hansen and Seufert 2003; Hellén et al. 2021; Pokorska et al. 2012). In our dataset, β_SQT_ tends to be higher in autumn and winter (Figure S5), when environmental stressors are milder and vegetation is not actively growing. Incorporating a seasonal β_SQT_ into model parameterizations remains challenging (Helmig et al. 2013), yet the influence of spring recovery on both temperature responses and SQT composition underscores the important role of biotic functions in regulating emissions. Better accounting for these seasonal variations in β_SQT_ could improve model representations of SQT fluxes, particularly in ecosystems where phenological shifts strongly modulate emission dynamics (Satake et al. 2024).

While temperature is the primary abiotic driver of sesquiterpene emissions, light plays a crucial role in regulating *de novo* synthesis, particularly for SQTs linked to current or recent photosynthetic activity (Duhl et al. 2008; Staudt et al. 2017). For instance, the emissions of β‐caryophyllene from orange trees have been shown to follow a light‐dependent pattern, indicating a direct influence of light availability on their biosynthesis rather than solely temperature‐driven volatilization (Hansen and Seufert 2003). A similar relationship was observed for β‐farnesene and bergamotenes emissions from pine trees, whereas α‐farnesene was emitted solely as a function of temperature (Geron et al. 2016). Sesquiterpene biosynthesis is influenced by compound‐specific properties, resulting in varied emission patterns and ecological functions, such as herbivore defense and pollinator attraction (Ghaffari et al. 2019; Holopainen et al. 2013). The complexity of light's influence is also evident in our review, with eleven studies reporting exclusively temperature‐driven emissions and four indicating regulation by both light and temperature. However, most studies did not control or measure light intensities, introducing further uncertainties regarding its role in regulating SQT emissions.

**FIGURE 2:**
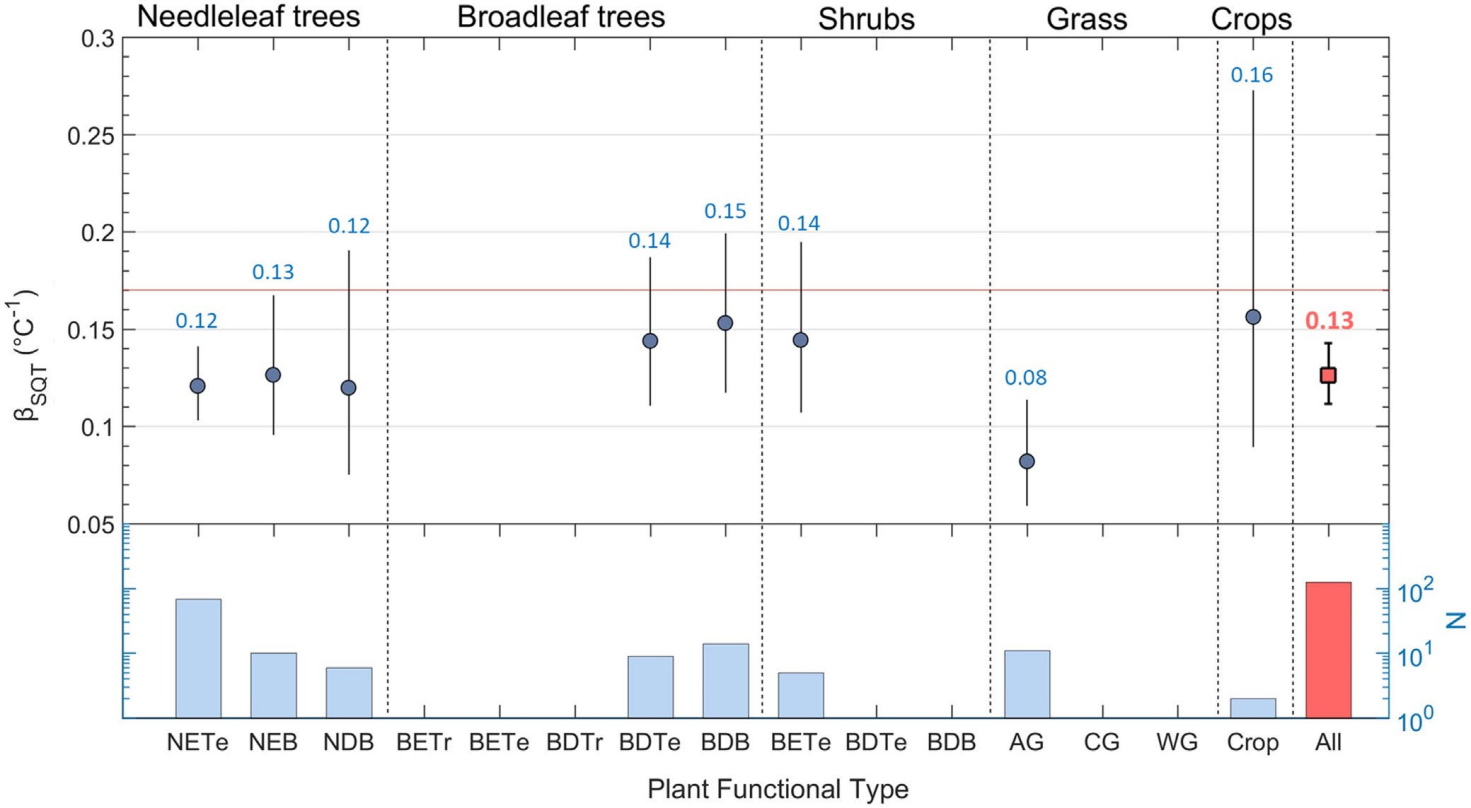
Temperature Sensitivities for Sesquiterpenes (β_SQT_) Across Plant Functional Types (PFTs). The error bars indicate 95% confidence intervals, and the size of the blue bullet points is proportional to the global surface area of each PFT. Abbreviations: NETe, needleleaf evergreen temperate forest; NEB, needleleaf evergreen boreal forest; NDB, needleleaf deciduous boreal forest; BETr, broadleaf evergreen tropical forest; BETe, broadleaf deciduous tropical forest; BDTe, broadleaf evergreen temperate forest; BDTs, broadleaf deciduous temperate shrubs; BDB, broadleaf deciduous boreal forest; AG, Arctic grass; CG, cool grass; WG, warm grass. The lowest part of the figure illustrates the number of samples (N) that were considered in the statistics.

Even if the interaction between biotic and abiotic factors shapes SQT emissions, refining Equation (1) by incorporating Plant Functional Types (PFTs) represents a key step forward. A meta‐analysis on monoterpene (MT) emissions, with six times the data availability, successfully applied machine learning to demonstrate PFT‐dependent temperature responses, demonstrating the viability of this approach (Bourtsoukidis et al. 2024). Similarly, an Analysis of Variance (ANOVA) test on SQT emissions reveals statistically significant differences (*p* < 0.001) among PFTs, with broadleaf species exhibiting greater temperature sensitivity than needleleaf species (Figure 2). This distinction aligns with the assumption that broadleaved plants, which rely more on de novo synthesis, respond more dynamically to short‐term temperature changes (Duhl et al. 2008). In contrast, needleleaf species tend to store SQTs in specialized resin ducts, buffering against immediate fluctuations and leading to lower β_SQT_ values (Staudt and Lhoutellier 2011). While regional characteristics, wider forest composition, and past climatic conditions can influence the temperature responses, integrating PFT‐dependent temperature sensitivities into global models considerably improves the accuracy of SQT emission predictions to better capture ecosystem‐specific dynamics.

### 2.2 Standardized Emission Rates for PFTs

Standardized emission potentials (*E*_*s*_) provide a uniform baseline for comparing SQT emissions across vegetation types, as implied in Equation (1). While β_SQT_ has often been assumed constant, it is well established that *E*_*s*_ varies significantly across PFTs. This variability is further influenced by physiological and environmental factors, including stress responses, acclimation, and developmental processes (Niinemets 2010; Niinemets et al. 2010). However, in the absence of broadly adopted emission inventories, these values remain relatively uniform in global emission models like MEGAN (Guenther et al. 2012) (Table S1), limiting their ability to accurately represent real‐world variability. The simplicity of empirical parameterizations stems from disregarding biochemical pathways, storage mechanisms, species‐specific adaptations, and environmental stresses, all of which interact dynamically (Peñuelas and Staudt 2010). These factors not only govern sesquiterpene synthesis and storage but also regulate their release in response to environmental drivers, further complicating emission predictions.

Our dataset reveals that PFT‐based *E*_*s*_ values are considerably lower than those currently used in emission model simulations, with variations spanning orders of magnitude even within the same functional categories (Figure S6; Table S1). While resolving these differences would be ideal for improving emission predictions, data limitations remain a challenge. Nevertheless, refining Equation (1) with PFT‐specific values would provide a more accurate representation of real‐world emissions while maintaining a framework adaptable to future refinements.

## 3 Connecting Biosphere, Atmosphere and Environmental Change

### 3.1 Model Simulations of Sesquiterpene Emissions and Their Role in Atmospheric Chemistry

To assess the sensitivity of emissions to PFT‐based parameterization refinements and their respective atmospheric implications, we performed model simulations coupling MEGAN emissions with the Community Atmospheric Model version 6 with chemistry (CAM6) (Emmons et al. 2020) in the Community Earth System Model (CESM) (Hurrell et al. 2013) (see methods in SI). This fully coupled Earth System framework enables the integration of land‐atmosphere interactions, including atmospheric chemical and meteorological changes, which are essential for evaluating SQT emissions and their chemical and climatic impacts. For our simulations, we used the updated TS2 chemistry scheme (Schwantes et al. 2020) with a 1° resolution for the year 2012. We conducted four simulations, using the baseline one (Guenther et al. 2012) as a reference while systematically introducing PFT‐dependent refinements for only β_SQT_ (simulation 2), only E_s_ (simulation 3), and both parameters together (simulation 4), applying the values listed in Table S1.

**FIGURE 3:**
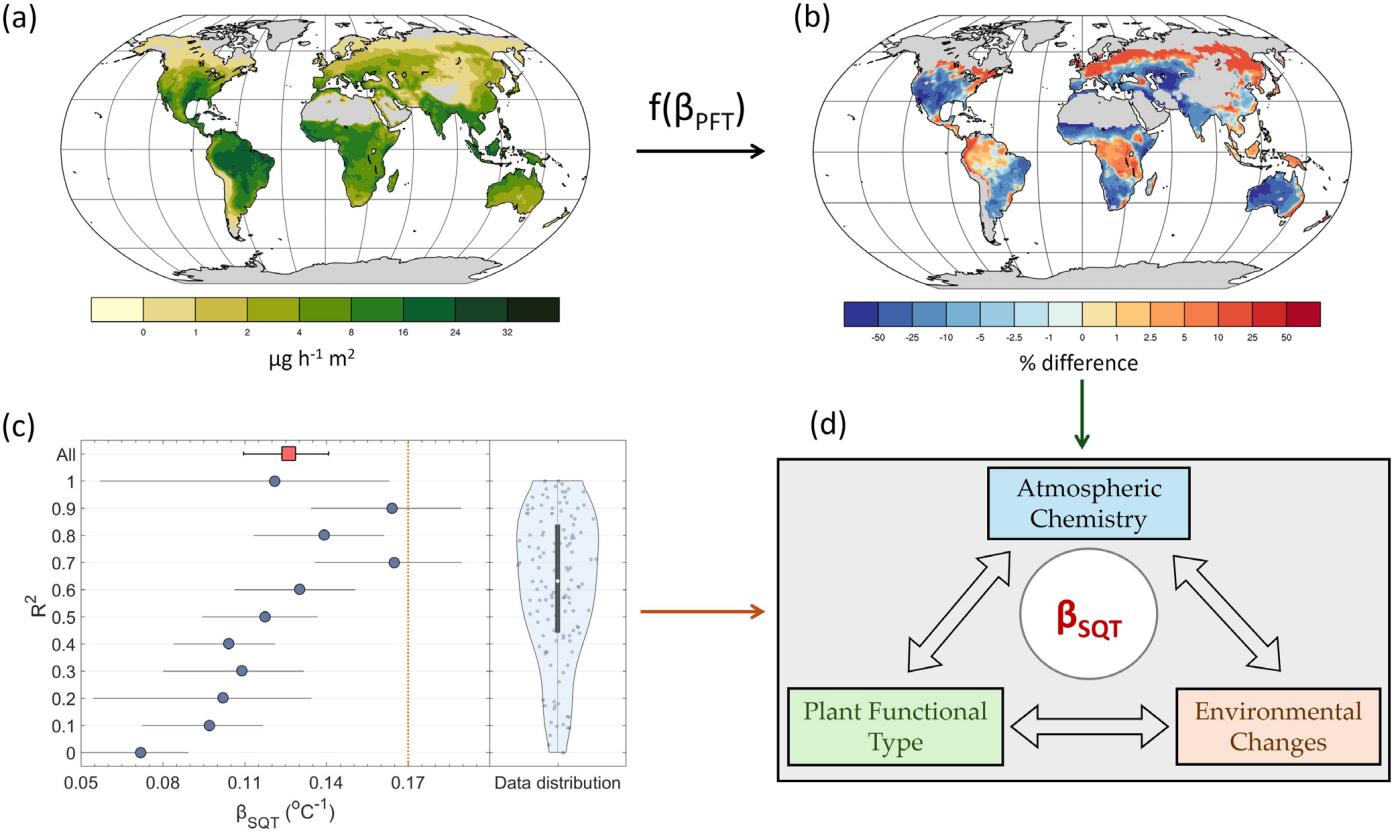
The Temperature Sensitivity of Sesquiterpene Emissions (β_SQT_) Mediates Interactions Between the Biosphere, Atmosphere, and Environmental Change. (a) Revised, plant functional type (PFT)‐dependent sesquiterpene emissions across global vegetation. (b) Percentage difference in sesquiterpene emissions utilizing a constant β_SQT_ value (0.17°C^−1^; indicated by the orange dashed line in c) compared to emissions as a function of PFT‐dependent temperature responses. (c) Groups of coefficients of determination (*R* ^2^) for experimentally derived dependencies of sesquiterpene emissions on temperature. (d) Conceptual “gray box” diagram of β_SQT_ being the regulator for underrepresented relationships over forest ecosystems.

By incorporating the statistical medians for PFT‐specific parameterizations into MEGAN, we estimate a global annual SQT emission of 15.6 Tg (Figure 3a). This value is nearly half of the previous estimate (29 Tg) (Guenther et al. 2012), primarily due to significantly lower emission potentials (E_s_) for the different PFTs. In these simulations, tropical and temperate forests emerge as the dominant sources of SQT emissions. However, given the absence of direct observations, the parameterization of emissions from tropical ecosystems remains largely speculative, underscoring the urgent need for more measurements to refine global estimates. SQTs undergo rapid oxidation by OH radicals, contributing to the missing OH reactivity observed in these ecosystems (Di Carlo et al. 2004; Nölscher et al. 2016; Pfannerstill et al. 2018). This oxidation influences the oxidative capacity of the atmosphere and methane lifetime, reinforcing the need to evaluate their cascading effects on atmospheric chemistry.

Focusing on temperature responses, we observe a mild reduction of approximately 24% on average (Figure 2), identifying a strong sensitivity in global emissions. Figure 3b illustrates the annual differences between the base simulation and simulation 2, showing that PFT‐dependent β_SQT_ leads to substantial positive and negative deviations. These differences become even more pronounced on seasonal and monthly scales, exceeding 100% in widespread ecosystems such as boreal and tropical forests (Figure S7).

The changes in SQT emissions could be further amplified by accounting for the tight mathematical coupling between β_SQT_ and *E*_*s*_ in Equation (1). Since β_SQT_ governs the exponential temperature scaling of emissions, even small uncertainties in its value can cascade into *E*_*s*_ estimates, introducing systematic biases. For instance, a common simplification across literature is to fix β_SQT_ at 0.17°C^−1^ and derive *E*_*s*_ accordingly. Consequently, biases in β_SQT_ directly translate into compensatory errors in *E*_*s*_, distorting parameterization outputs and reinforcing the need to refine both parameters simultaneously to enhance model accuracy and better capture real‐world variability.

To assess the impact of simultaneous PFT‐based refinements on the global atmospheric chemistry implications, we calculated the mean relative annual differences in surface ozone and SOA (simulation 4). Surface ozone mixing ratios increased over tropical forests and their downwind outflow regions, with the most pronounced enhancement observed over the Amazon rainforest and East Africa (Figure S8). This pattern highlights the role of SQTs in reacting with NO_x_ over the NOx‐limited forested regimes, leading to ozone production in regions distant from the primary emission source (Finlayson‐Pitts and Pitts 1997). Given that background ozone levels over pristine tropical forests, such as the Amazon, typically range between 0.5 and 10 ppb during dry seasons (Andreae et al. 2015), the simulated increases represent a considerable change in atmospheric chemistry, particularly at finer temporal scales where peak deviations are more pronounced.

Similarly, our simulations indicate a considerable reduction in SOA concentrations over tropical forests, with annual reductions reaching up to 4 μg/m^3^ in localized regions of the Amazon. Observational studies and model simulations suggest that biogenic SOA dominates organic aerosol mass in the Amazon, with concentrations within the same range (Hodzic et al. 2016; Spracklen et al. 2011). Recent findings show that biogenic precursors, such as isoprene and its oxidation products, significantly contribute to SOA formation at higher altitudes, influencing aerosol growth and composition (Curtius et al. 2024). The magnitude of these changes underscores the critical role of more reactive BVOC emissions, such as SQTs, in atmospheric chemistry and physics. Accurately parameterizing these emissions is essential for improving SOA formation predictions and understanding their implications for aerosol‐cloud interactions, ultimately influencing the radiative balance and regional climate.

### 3.2 Environmental Changes as an Overlooked Mediator in Biosphere‐Atmosphere Interactions

Temperature remains the primary control over SQT emissions, but its apparent influence is not uniform across all observations. The strength of this relationship varies, reflecting the degree to which other environmental drivers modulate emission responses. As seen in Figure 3c, the positive correlation between β_SQT_ and *R* ^2^ (derived from the regression fits of Equation (1) on the experimental data considered in this meta‐analysis) indicates that temperature sensitivity strengthens when emissions are predominantly driven by temperature. Higher *R* ^2^ values correspond to higher β_SQT_, suggesting that in conditions where temperature is the primary controlling factor, SQT emissions respond more dynamically. Conversely, at mid‐to‐lower *R* ^2^ values, β_SQT_ decreases, implying that additional environmental factors, such as biotic and/or abiotic factors, contribute to emission variability, diminishing the direct influence of temperature.

Consequently, the temperature sensitivity of sesquiterpene emissions (β_SQT_) emerges as a dynamic mediator at the interface of plant functional types, atmospheric chemistry, and environmental change (Figure 3d). Traditionally regarded as a simple scaling factor governing emission responses to temperature, our synthesis suggests that β_SQT_ encompasses far more than just a mechanistic parameterization. Instead, it reflects the intricate interplay between biological processes and atmospheric composition, responding not only to temperature fluctuations but also to ecosystem‐level stressors and physiological acclimation.

Given its responsiveness to environmental variability, we propose that β_SQT_ can serve as a “volatile stressometer”, representing an empirical proxy that links biosphere‐atmosphere interactions to environmental stressors. Incorporating this variability into global models would allow for a more dynamic representation of BVOC emissions, shifting away from fixed parameterizations toward stress‐responsive formulations that better capture plant‐environment interactions. Accounting for species‐specific acclimation, biotic triggers, and multi‐stressor responses could significantly enhance emission modeling accuracy, particularly in regions experiencing rapid environmental shifts and extreme weather events. Generally, this conceptual framework underscores the need for a more integrated approach to BVOC research, where plant functional types, atmospheric chemistry, and environmental changes are treated as interconnected components rather than isolated factors.

## 4 A Surprising Decline in Temperature Sensitivity Across Time

**FIGURE 4:**
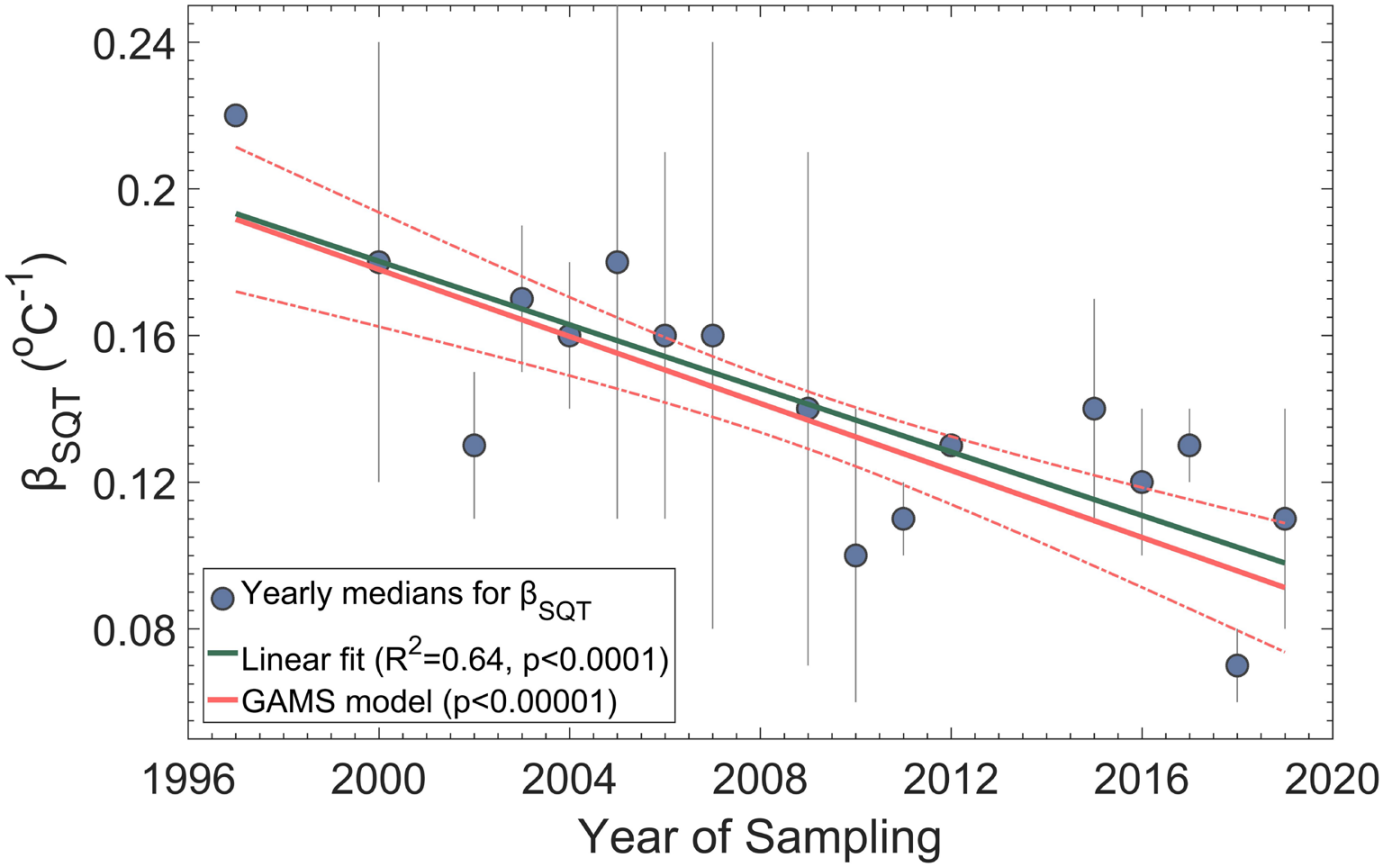
Temporal Decline in Temperature Responses for Sesquiterpene Emissions (β_SQT_) Over the Years. The blue circles represent the yearly median β_SQT_ values, with error bars indicating the 95% confidence intervals. The green line shows the linear fit of the median values, described by the equation: β_SQT_ = −0.0043Yr + 8.8. The thick red line represents the results of Generalized Additive Models (GAMs), with the upper and lower limits illustrated by red dashed lines.

The accelerating pace of environmental change imposes increasing stresses on ecosystems, altering plant physiological responses in ways that remain only partially understood. Rising global temperatures, drought, increasing atmospheric CO_2_ levels, ground level ozone, land use changes, and shifting precipitation patterns have been shown to influence vegetation functioning at multiple scales (Calvin et al. 2023; Cramer et al. 2001; Heald and Spracklen 2015; Lin et al. 2020; Otu‐Larbi et al. 2020; Peñuelas and Staudt 2010). In line with these shifts, we identify a statistically significant decline in β_SQT_ over the past two decades (Figure 4). Linear regression analysis reveals a robust negative trend (*R* ^2^ = 0.64, *p* < 0.0001), demonstrating a systematic weakening of temperature‐driven emission responses over time. This decline is further corroborated by a more flexible, non‐parametric approach using Generalized Additive Models (GAMs) (Wood 2017; Yee and Mitchell 1991), which accounts for potential nonlinearities while reinforcing the statistical significance of the trend (*p* < 0.00001). The strong negative association between β_SQT_ and sampling year suggests that environmental pressures, rather than random variation, are driving a fundamental shift in how ecosystems regulate emissions.

A key consideration is whether this decline reflects a genuine shift in temperature responses or is a result of methodological biases introduced by improved measurement techniques in recent years. However, multiple lines of evidence suggest the shift is genuine. Gas chromatography and offline adsorption tube analyses, the dominant methods for sesquiterpene quantification (Figure S10), have remained the gold standard for decades, ensuring methodological consistency across studies. Additionally, *R* ^2^ values have also declined significantly over time (*p* < 0.001), indicating weaker relationships between temperature and sesquiterpene emissions in more recent years. This suggests that non‐temperature‐related environmental stressors are increasingly governing SQT emissions, reducing their direct dependence on temperature.

Moreover, if this trend was purely driven by methodological biases, a similar decline in temperature sensitivity would be expected for monoterpenes (MTs), yet such a pattern was not observed in the respective meta‐analysis (Bourtsoukidis et al. 2024). The comparison between β_SQT_ and β_MT_ reveals that, while SQTs generally exhibit higher temperature sensitivities, their correlation has weakened in recent years (Figure S11). This may be attributed to biological factors; however, methodological advancements in both sampling setups and analytical approaches cannot be entirely ruled out. Sesquiterpene measurements have historically encountered greater methodological challenges than those for monoterpenes, primarily due to their increased susceptibility to mechanical stress, higher reactivity, stronger surface affinity, and broader chemical diversity. These challenges underscore the importance of recent improvements in SQT detection when interpreting long‐term trends. While this complexity introduces some degree of uncertainty, the observed decline remains statistically significant across various methodologies and ecosystems.

Taken together, it appears that the declining β_SQT_ reflects broader ecosystem‐level changes. Whether these changes stem from long‐term acclimation, physiological trade‐offs, or shifts in biochemical allocation remains an open question. Our findings challenge the notion of β_SQT_ as a static parameter, revealing it instead as a dynamic variable that evolves alongside environmental change. This realization not only challenges traditional emission models but also underscores the need to integrate adaptive vegetation responses into atmospheric chemistry frameworks.

## 5 Summary and Future Directions

Although temperature is the principal abiotic factor for shaping sesquiterpene emissions, several other environmental variables can modulate both emission magnitudes and their relationship to temperature. Elevated CO_2_ alters carbon allocation in plants, influencing terpene biosynthesis and storage pools, and hence, SQT emissions (Duhl et al. 2008; Fares et al. 2011; Ghirardo et al. 2011; Guenther et al. 2006). Soil moisture extremes, whether drought or flooding, can similarly induce stress‐driven changes, either by depleting internal reserves or triggering abrupt emission bursts (Bourtsoukidis et al. 2014a; Hansen and Seufert 2003; Hellén et al. 2021; Seco et al. 2015). Under extreme heat events, plants may temporarily decouple their temperature‐emission response as they switch from typical growth processes to protective or survival modes (Birami et al. 2021; Joó et al. 2011; Nagalingam et al. 2022, 2023; Staudt and Lhoutellier 2011). Ozone pollution adds further complexity, as moderate ozone levels can stimulate protective sesquiterpene production (Bourtsoukidis et al. 2012; Kanagendran et al. 2018; Ossola and Farmer 2024; Yang et al. 2025), whereas high ozone concentrations can impair stomatal function and degrade SQTs near the leaf boundary layer and inside the forest canopy (Calfapietra et al. 2016; Fares et al. 2010; Jardine et al. 2011; Niinemets 2002; Ossola and Farmer 2024). More broadly, air pollution alters plant morphoanatomical and physiological responses (De Araújo et al. 2025), introducing further abiotic stressors that remain largely underexplored. These challenges can be particularly pronounced in urban environments, where SQT emissions not only contribute to air quality dynamics (Calfapietra et al. 2013; Fitzky et al. 2019; Ghirardo et al. 2016; Pfannerstill et al. 2024) but are also subject to air and soil pollution feedbacks that could modify their biotic functions in ways that are yet to be fully understood.

Biotic factors exert critical, often underrecognized, controls on sesquiterpene emissions. Seasonal progressions can strongly modulate SQT fluxes, with pronounced increases before leaf senescence or during needle expansion (Bourtsoukidis et al. 2014b; Helmig et al. 2013), while episodic stresses such as pathogens or herbivore outbreaks prompt sudden and substantial emission spikes (Bourtsoukidis et al. 2014b; Kajos et al. 2013; Matsunaga et al. 2011). In addition, transient bursts of ecologically specialized SQT structures can arise during pollination events, linking chemical signaling to reproductive success (Haapanala et al. 2009; Stirling et al. 2024). Plants often “remember” previous stimuli, so prior stress exposure can enhance or diminish subsequent emission responses (Bruce et al. 2007; Helmig et al. 2007; Pokorska et al. 2012). Collectively, these diverse biological triggers underscore that SQT production is not merely a passive function of abiotic variables, but a dynamic outcome of plant defense strategies, developmental processes, evolutionary adaptation, and diverse ecological interactions.

Despite being studied separately, biotic and abiotic drivers frequently co‐occur in natural ecosystems, exerting interactive effects on SQT emissions. For instance, heat and drought stress often coincide with herbivore outbreaks or pathogen invasions, amplifying emission responses in ways that differ from individual stressors alone (Catola et al. 2018; Copolovici et al. 2014; Weldegergis et al. 2015). Similarly, air pollution (particularly ozone exposure) has been shown to alter plant chemical defenses, influencing how SQTs mediate ecological interactions such as pollination signals (Blande et al. 2014; Langford et al. 2023) and their overall contribution to OH reactivity and SOA formation (Bonn 2003; Bonn et al. 2014; Dada et al. 2023; Nölscher et al. 2013). Moreover, some sesquiterpenes may be predominantly regulated by temperature, while others are more closely associated with stress‐induced processes. Consequently, total SQT emission responses are likely to reflect a combination of these mechanisms, making their speciation critical for understanding emission dynamics under varying environmental conditions. However, these interactions remain challenging to parameterize, as the complexity of their combined effects complicates their representation in global models, particularly given that these phenomena occur at small temporal and spatial scales.

The current literature remains heavily skewed toward short‐term, single‐driver experiments, which often fail to capture the full complexity of interlinked emission responses. While controlled studies have elucidated fundamental processes, they are limited and rarely reflect the dynamic stress interactions observed in natural ecosystems (Bourtsoukidis et al. 2024). Additionally, most field studies are geographically biased, with an overrepresentation of temperate and boreal ecosystems, leaving urban, agricultural, tropical, and arid biomes largely unexamined. This divergence limits direct comparability across datasets, introducing uncertainties in meta‐analyses such as ours and calls for more observations at the interface of environmental and ecological stresses.

Bridging these knowledge gaps requires a shift toward integrated, long‐term observational networks that capture emission variability across diverse environmental conditions. Large‐scale field studies in underrepresented ecosystems, particularly tropical and arid regions, are crucial for refining global emission models. Additionally, multi‐factorial experiments that simultaneously manipulate biotic and abiotic stressors will provide key insights into their interactive effects on emissions. Controlled experiments simulating future climate scenarios such as extreme drought, heat stress, and elevated CO_2_ could further elucidate the physiological trade‐offs of SQT emissions. Perhaps the most pressing need is to establish quantitative thresholds for heat and drought stress tipping points, identifying the conditions under which emission dynamics shift from resilience to vulnerability, triggering cascading effects on atmospheric chemistry. Addressing these uncertainties is particularly crucial as climate extremes intensify, increasing the likelihood of prolonged stress events that alter biosphere‐atmosphere feedbacks.

In conclusion, the temperature sensitivity of sesquiterpene emissions (β_SQT_) is not a fixed parameter, but a dynamic trait shaped by environmental complexity. While temperature remains a key driver, our synthesis reveals that β_SQT_ responds to a broader suite of biotic and abiotic stressors, challenging the way BVOC emissions are currently parameterized. The observed long‐term decline in β_SQT_ suggests that shifting environmental pressures are altering plant–atmosphere interactions in ways that remain largely unquantified. As ecosystems face global change, β_SQT_ may serve as an empirical “volatile stressometer”, capturing plant stress responses with direct implications for atmospheric chemistry. Characterizing this adaptive variability is critical for improving emission models, bridging ecological and atmospheric sciences, and ultimately refining our predictions of biosphere–atmosphere feedbacks in a changing world.

## Author Contributions

**Efstratios Bourtsoukidis:** conceptualization, data curation, formal analysis, investigation, methodology, visualization, writing – original draft. **Alex Guenther:** formal analysis, investigation, methodology, resources, software, writing – review and editing. **Hui Wang:** formal analysis, software, writing – review and editing. **Aliki Christodoulou:** data curation, validation, writing – review and editing. **Theo Christoudias:** visualization, writing – review and editing. **Anke Nölscher:** data curation, writing – review and editing. **Ana M. Yañez‐Serrano:** data curation, validation, writing – review and editing. **Josep Peñuelas:** validation, writing – review and editing.

## Conflicts of Interest

The authors declare no conflicts of interest.

## Supporting information


Data S1.

## Data Availability

The meta‐analysis dataset is available at: https://doi.org/10.5281/zenodo.15274959.
