# Supplementary material for: Environmental Change Is Reshaping the Temperature Sensitivity of Sesquiterpene Emissions and Their Atmospheric Impacts
Source: Glob Chang Biol. 2025 Jun 4;31(6):e70258. doi: 10.1111/gcb.70258 (PMC12135033; doi:10.1111/gcb.70258)
Supplement: Supplementary file 1 — Data S1. [file GCB-31-e70258-s001.pdf]

Supporting Information for:

Environmental Change is Reshaping the Temperature Sensitivity of Sesquiterpene Emissions and Their Atmospheric Impacts

Efstratios Bourtsoukidis[1], Alex Guenther[2], Hui Wang[2], Theo Economou[1,3], Georgia Lazoglou[1], Aliki Christodoulou[1,a], Theo Christoudias[1], Anke Nölscher[4], Ana M. Yañez-Serrano[5], and Josep Peñuelas[6,7]

[1]Climate and Atmosphere Research Center (CARE-C), The Cyprus Institute, Nicosia, Cyprus
[2]Department of Earth System Science, University of California, Irvine, USA
[3]Department of Mathematics and Statistics, University of Exeter, Exeter, UK
[4]Bayreuth Center of Ecology and Environmental Research, University of Bayreuth, Bayereuth, Germany
[5]Institute of Environmental Assessment and Water Research (IDAEA), Barcelona, Spain
[6]Center for Research Ecology and Forestry Applications (CREAF), Bellaterra, Barcelona, Spain
[6]Global Ecology Unit CREAF-CSIC-UAB, Bellaterra, Barcelona, Spain
[a] Now at: PSI Center for Energy and Environmental Sciences, Villigen, Switzerland

Correspondence: Efstratios Bourtsoukidis (e.bourtsoukidis@cyi.ac.cy)

The document includes:

Methods

Supporting Figures: 11

Supporting Tables: 1

S1 | Methods

S1.1 | Data Collection and Vectorization

We conducted a systematic search of the Web of Science (WoS), including the WOS, BCIBIOSIS, CCC, DRCI, and RSCI databases, using the keywords “sesquiterpenes,” “emissions,” and “temperature.” This search identified 146 peer-reviewed articles published between 2001 and 2021. Following a detailed screening process, 24 studies met our inclusion criteria by reporting regression-based temperature sensitivities (β values) derived from observational data. In total, 139 β coefficients were extracted.

For each observation, we compiled a suite of contextual variables, resulting in 20 annotated parameters per β value. These parameters included geographic information (latitude, longitude, elevation), experimental conditions, plant species and their classification, plant functional type (PFT), seasonal context, and key statistical metrics such as the coefficient of determination (R^2). Where categorical or textual information was reported, we converted it into numerical values for statistical analysis. All variables are included in the accompanying open-access dataset.

The studies employed diverse experimental methods, including variations in sampling strategies (enclosures, eddy covariance, online, offline), VOC quantification methods (Gas Chromatography, Mass Spectrometry), and environmental controls (laboratory, field measurements). Each β value was annotated according to the dominant method used in the respective study. In cases where multiple techniques were applied, we selected the method most relevant to the reported β coefficient. When specific sampling dates or geographic coordinates were unavailable, we derived these using information from cited literature or geographic tools such as Google Earth to maintain consistency across observations.

Plant species were categorized into PFTs following the MEGAN model classification. While some ecological attributes (such as tree height or age) were inconsistently reported, core spatial and biological metadata were collected for all selected studies. This thorough annotation process enabled robust cross-study comparisons in the meta-analysis.

S1.2 | Random Effect Statistics

To enhance the robustness of our meta-analysis, we excluded extreme values by retaining βSQT estimates within the 5th to 95th percentile range. This filtering resulted in a curated dataset of 125 observations, preserving representative variability while minimizing statistical noise. Observations that did not report an R^2 value were excluded from the respective analyses.

To address variability among studies, we adopted a linear mixed effects modeling approach previously developed for monoterpenes (Bourtsoukidis et al., 2024). We first fitted a baseline model without predictors to estimate the global mean of βSQT across the dataset. Next, we included PFT as a categorical fixed effect to assess variations across vegetation classes. To investigate the influence of regression quality, we incorporated R^2 as a covariate, categorized into intervals of 0.1, and examined its effect under both field and controlled conditions.

All models included a random intercept for study identity to account for data clustering and between-study variability. Model fitting was performed using the lmer function from the lme4 package in R (version 4.1.2), applying restricted maximum likelihood (REML) estimation. Uncertainty in model estimates was assessed using 95% confidence intervals.

S1.3 | Generalized Additive Models (GAMs)

Generalized Additive Models (GAMs) were employed to explore potential non-linear trends in β_{SQT} over time. Unlike standard linear models, GAMs relax the assumption of a strictly linear relationship between the year of observation and β_{SQT}. This flexibility allows the model to adapt to the data structure while still retaining the possibility of a linear fit if supported by the evidence.

We implemented GAMs using the mgcv package in R, following the methodology described by Wood (2017). In this framework, the mean of β_{SQT} is modeled as:

$$E(\beta_{SQT}) = f(\text{year}) + \alpha$$

where f(year) represents a smooth function constructed using thin-plate regression splines, and α is the intercept. The model is estimated using restricted maximum likelihood (REML), which objectively penalizes excessive flexibility in the smooth term. This penalization process minimizes out-of-sample predictive error, ensuring that the fitted function is neither overfit ("too wiggly") nor restricted to linearity unless clearly justified by the data. In doing so, GAMs provide a data-driven approach to capture potential non-linear temporal dynamics in β_{SQT}.

S1.4 | Global Model Simulations

The model simulations were conducted by coupling emissions from the Model of Emissions of Gases and Aerosols from Nature (MEGAN) with the Community Atmospheric Model version 6 with chemistry (CAM6), integrated within the Community Earth System Model (CESM) framework.

For our study, we employed the updated TS2 chemistry scheme with a 1-degree spatial resolution and conducted simulations specifically for the year 2012. All analyses presented here are based on annual mean outputs from this single-year simulation. CAM6 simulations, as conducted here, are deterministic in nature and do not incorporate internal variability or stochastic processes. Therefore, the reported differences among simulations directly illustrate the model’s sensitivity to variations in the prescribed parameters (β_{SQT} and Es) rather than reflecting random variability or uncertainty. While the current resolution captures global-scale atmospheric patterns, it may not adequately represent regional processes such as complex terrain-driven transport or topography-induced circulation, which can influence regional chemical distributions. Moreover, meteorological variability specific to the simulation year (2012) may have amplified certain features. Future work employing higher spatial resolution and multi-year simulations would allow for a more detailed understanding of regional transport dynamics and their effects on surface ozone.

The PFT-specific values were compiled directly from literature reports. For PFTs lacking direct observations, we estimated representative values by averaging those from closely related,

observationally constrained PFTs. For example, in the absence of data for Broadleaf Evergreen Tropical Trees (BETr), we approximated β_{SQT} and Es values using available measurements from other broadleaf tree categories, namely Broadleaf Deciduous Temperate Trees (BDTe) and Broadleaf Deciduous Boreal Trees (BDB). Given the large geographic distribution of BETr, this approach inevitably introduces substantial uncertainties into our revised global emissions. Consequently, addressing this knowledge gap through targeted observational studies, especially in tropical ecosystems, is essential to improving global sesquiterpene emission estimates and accurately quantifying their atmospheric impacts.

References

Bourtsoukidis, E., Pozzer, A., Williams, J., Makowski, D., Peñuelas, J., Matthaios, V. N., Lazoglou, G., Yañez-Serrano, A. M., Lelieveld, J., Ciais, P., Vrekoussis, M., Daskalakis, N., & Sciare, J. (2024). High temperature sensitivity of monoterpene emissions from global vegetation. *Communications Earth & Environment*, *5*(1), 23. https://doi.org/10.1038/s43247-023-01175-9

Wood, S. N. (2017). *Generalized Additive Models: An Introduction with R* (2nd ed.). Chapman and Hall/CRC. https://doi.org/10.1201/9781315370279

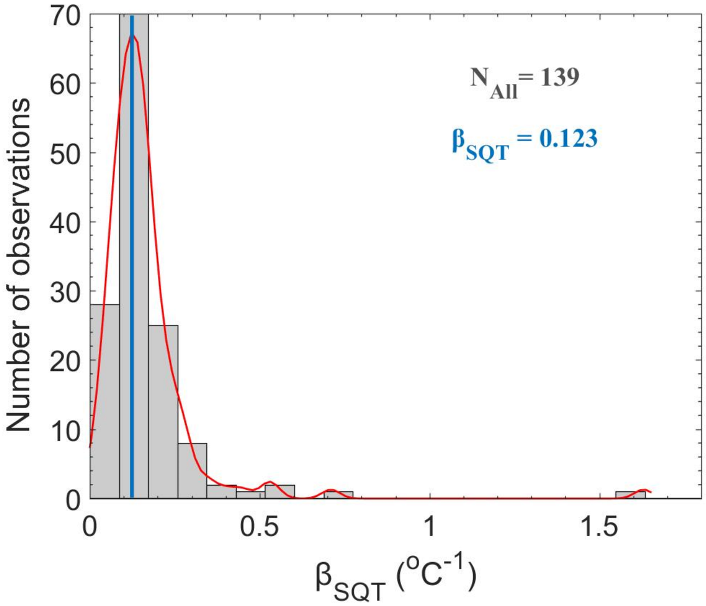

Figure S1 | Histogram of all reported β_{SQT} values. The red curve represents a kernel density estimate (KDE), with the peak indicating the central tendency of the β_{SQT} distribution.

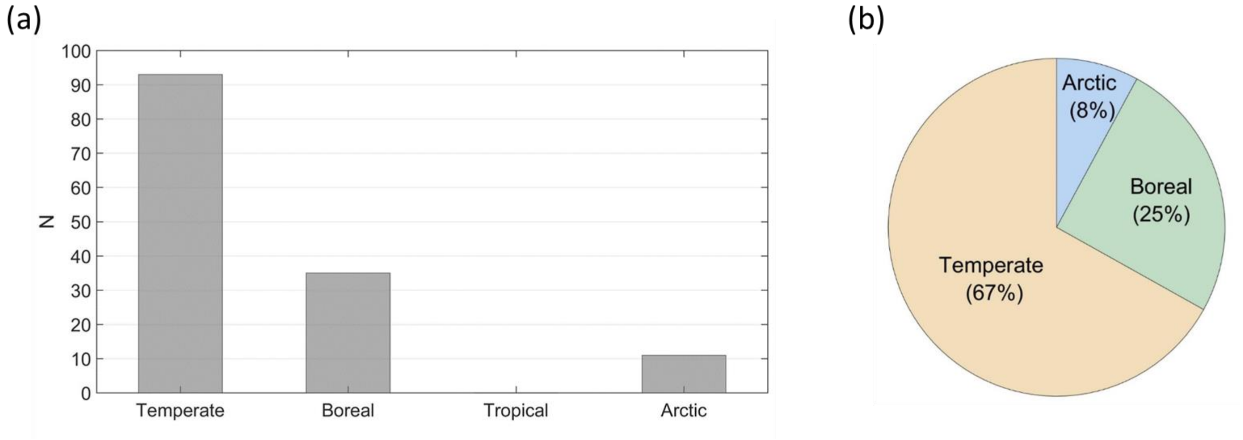

Figure S2 | Number of Temperature Sensitivities for Sesquiterpenes (β_{SQT}) Collected Across Different Ecosystems. (a) Total number (N) of β_{SQT} values across the four dominant ecosystems. (b) Percentage (%) distribution of samples collected from each ecosystem.

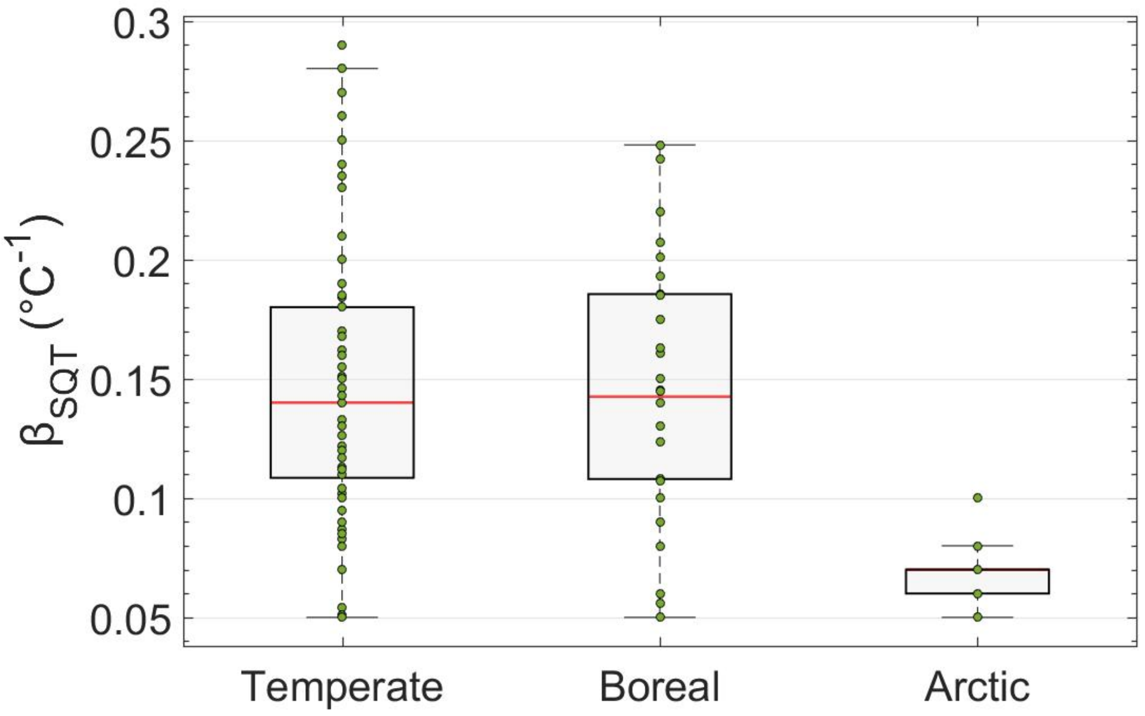

Figure S3 | Ecosystem Variability in β_{SQT} Boxplot Statistics. Each boxplot shows the distribution of β_{SQT} values for a given ecosystem. The red line denotes the median, while the box boundaries indicate the 25th and 75th percentiles. Whiskers extend to the most extreme data points not classified as outliers. Green circles represent individual data points included in the analysis for each ecosystem.

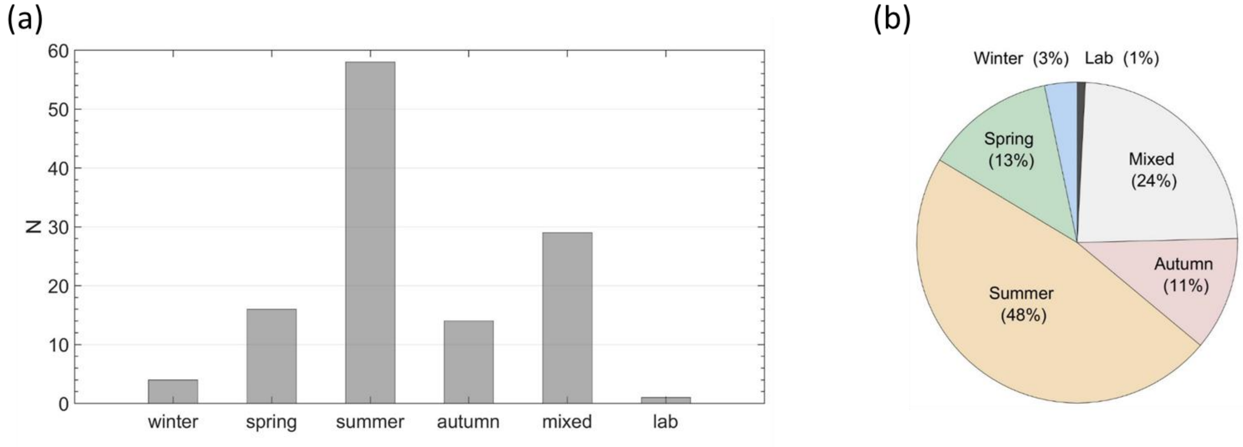

Figure S4 | Number of Temperature Sensitivities for Sesquiterpenes (β_{SQT}) Collected Across Different Seasons. (a) Total number of β_{SQT} values, where "mixed" indicates measurements spanning multiple seasons, and "lab" refers to laboratory-based observations. (b) Percentage (%) distribution of samples collected across each season.

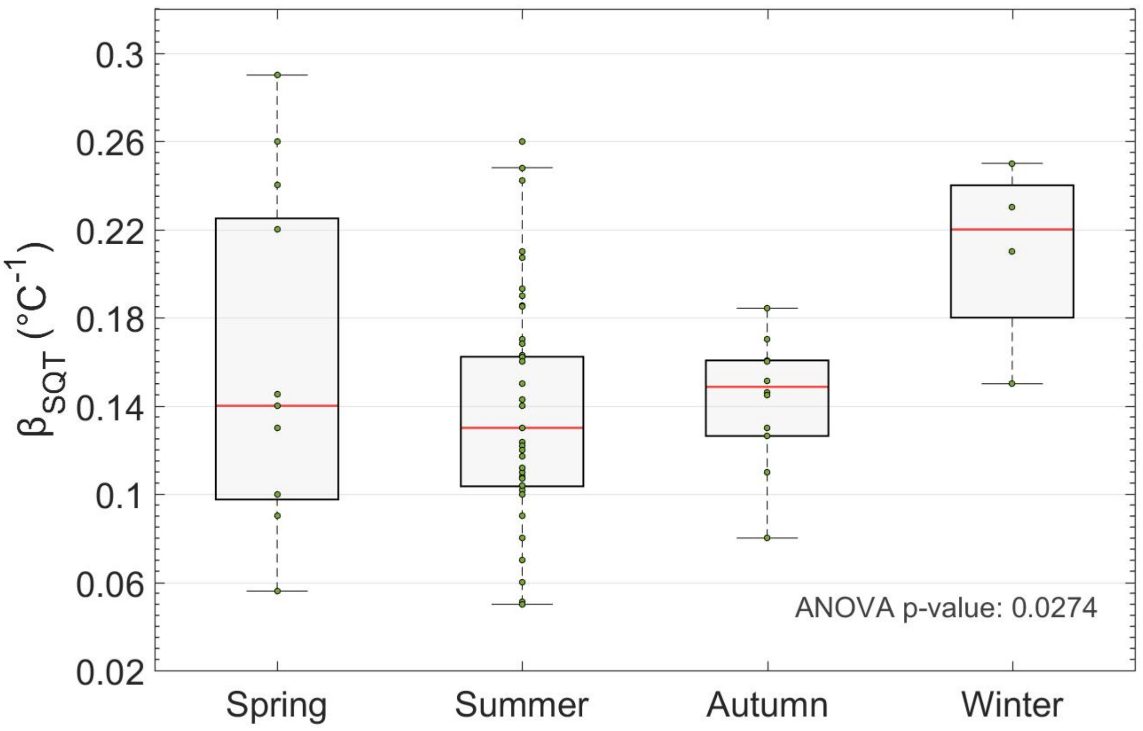

Figure S5 | Seasonal Variability in β_{SQT} Boxplot Statistics. Each boxplot shows the distribution of βSQT values for a given season. The red line denotes the median, while the box boundaries indicate the 25th and 75th percentiles. Whiskers extend to the most extreme data points not classified as outliers. Green circles represent individual data points included in the analysis for each season.

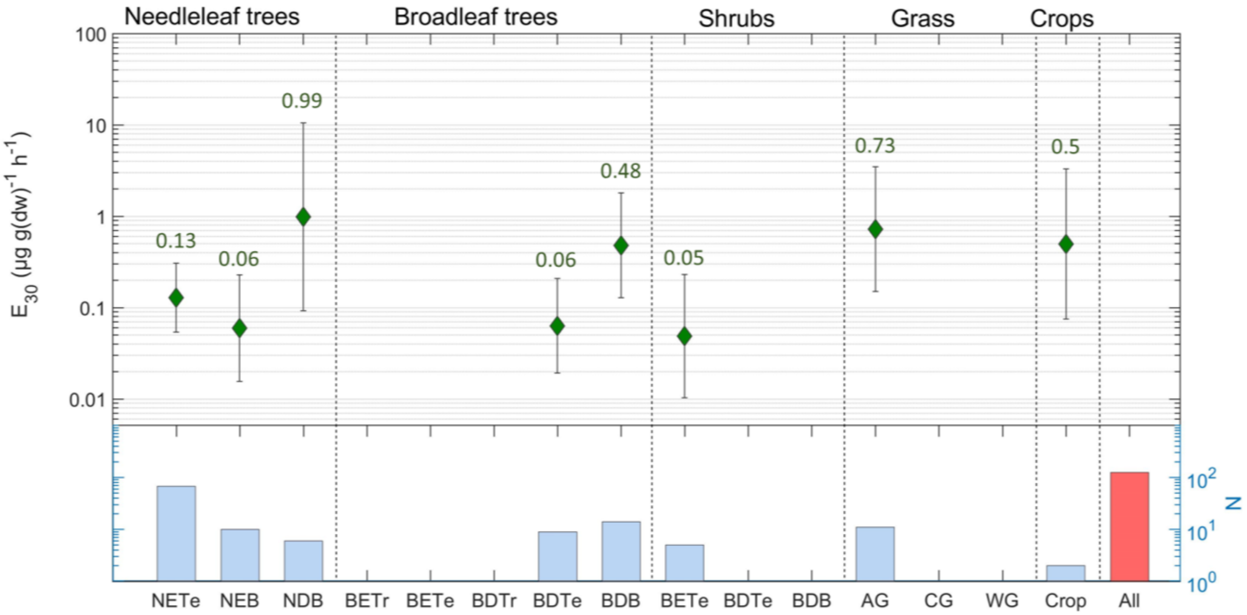

Figure S6 | Standardized Emission Potential at 30ºC Across Plant Functional Types (PFTs). The error bars indicate 95% confidence intervals, and the size of the blue bullet points is proportional to the global surface area of each PFT. Abbreviations: NETe, needleleaf evergreen temperate forest; NEB, needleleaf evergreen boreal forest; NDB, needleleaf deciduous boreal forest; BETr, broadleaf evergreen tropical forest; BETe, broadleaf deciduous tropical forest; BDTe, broadleaf evergreen temperate forest; BDTs, broadleaf deciduous temperate shrubs; BDB, broadleaf deciduous boreal forest; AG, Arctic grass; CG, cool grass; WG, warm grass.

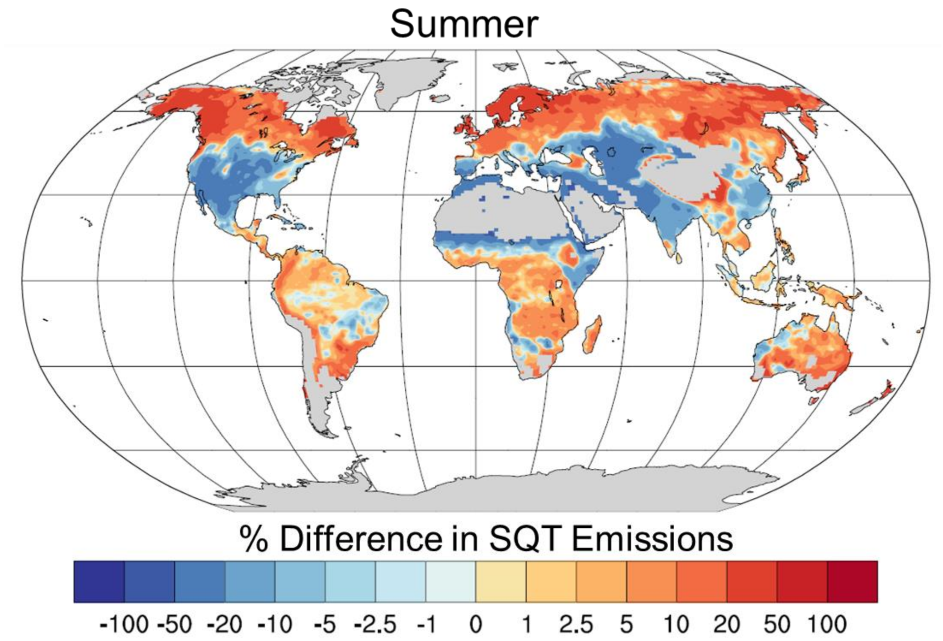

Figure S7 | Summertime Differences in Sesquiterpene Emissions. The differences represent the deviation between the base simulation, which assumes a constant β_{SQT} value (0.17 °C^{-1}), and emissions modeled as a function of PFT-dependent temperature responses.

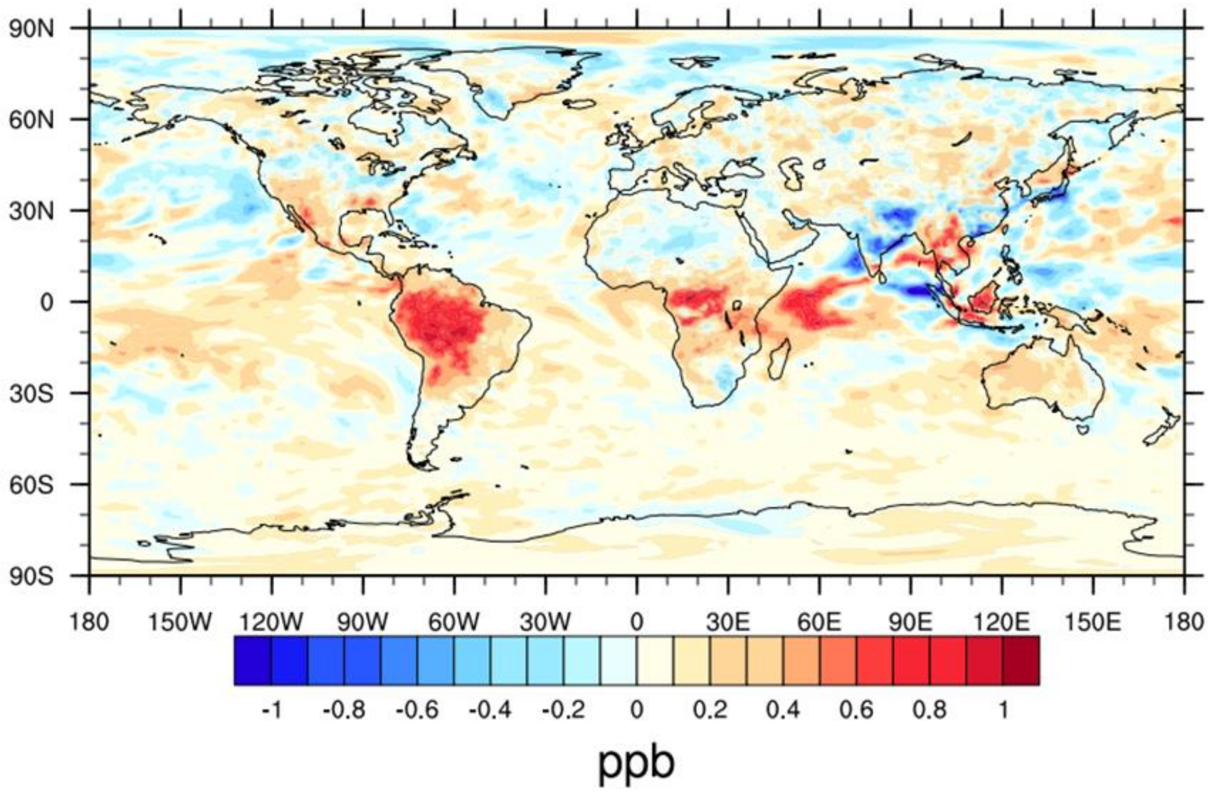

Figure S8 | Mean Relative Annual Differences in Surface Ozone. The differences are calculated between a constant temperature sensitivity parameterization (β_{SQT} = 0.17) and a PFT-dependent parameterization. The values used for this analysis are derived from Figures S6 and S7.

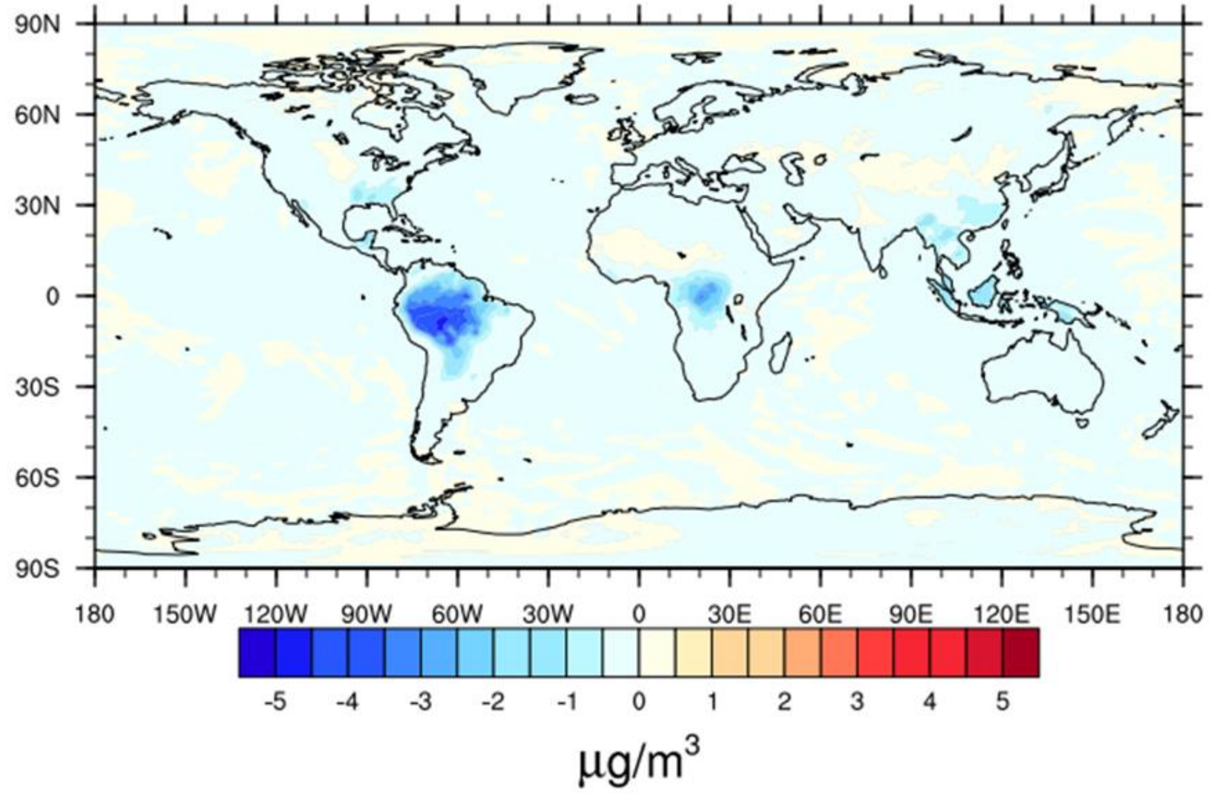

Figure S9 | Mean Relative Annual Differences in Secondary Organic Aerosol (SOA). The differences are calculated between a constant temperature sensitivity parameterization (β_{SQT} = 0.17) and a PFT-dependent parameterization. The values used for this analysis are derived from Figures S6 and S7.

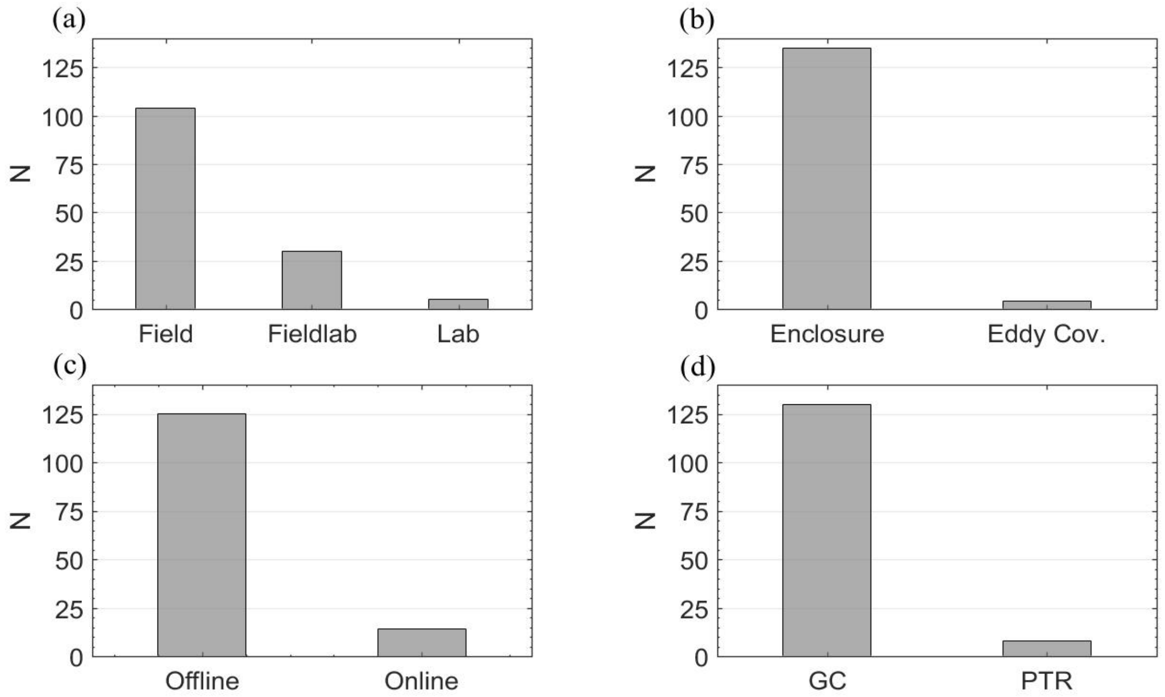

Figure S10 | Number of Temperature Sensitivities for Sesquiterpenes (βSQT) Collected Across Different Experimental Methodologies. (a) Environmental conditions under which βSQT values were derived (FieldLab refers to plants re-potted in a field environment distinct from their original habitat). (b) Methods used for determining emission rates, with "Enclosure" referring to individual plants and "Eddy Covariance" representing whole ecosystems. (c) Techniques employed for VOC sampling, categorized as offline or online methods. (d) Instrumentation used for VOC analysis, including Gas Chromatography (GC) and Proton Transfer Reaction Mass Spectrometry (PTR-MS; encompassing both Time-of-Flight (ToF) and Quadrupole systems).

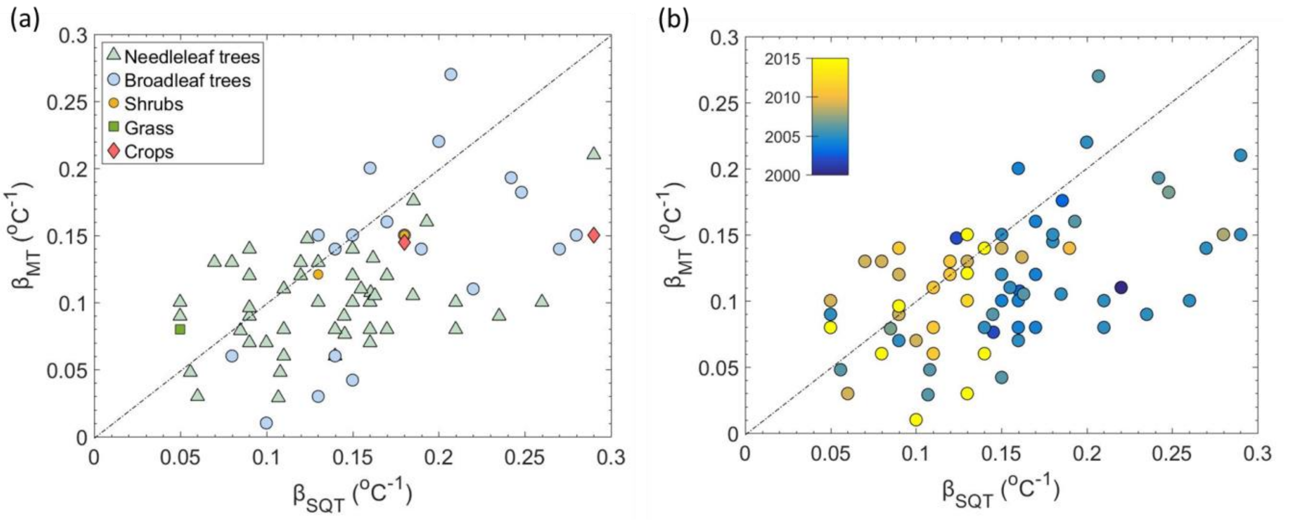

Figure S11 | Relationship between the Temperature Sensitivity of Sesquiterpenes (β_{SQT}) and Monoterpenes (β_{MT}). (a) Data clustered by broader plant functional type (PFT) categories. (b) Data color-coded by the year of observations.

Plant Functional Type (PFT)	Default β_{SQT} ($^{o}C^{-1}$)	Revised β_{SQT} ($^{o}C^{-1}$)	Default Es ($\mu g\ m^{2}\ h^{1}$)	Revised Es ($\mu g\ m^{2}\ h^{1}$)
NET temperate	0.17	0.121	240	27.1
NET boreal	0.17	0.127	240	12.5
NDT boreal	0.17	0.120	240	89.0
BET tropical	0.17	0.149	240	27.2
BET temperate	0.17	0.149	180	27.2
BDT tropical	0.17	0.149	240	21.8
BDT temperate	0.17	0.144	180	5.1
BDT boreal	0.17	0.153	180	38.5
BES temperate	0.17	0.144	190	6.4
BDS temperate	0.17	0.149	190	27.2
BDS boreal	0.17	0.149	190	27.2
C3 Arctic grass	0.17	0.082	6	43.5
C3 grass	0.17	0.082	6	43.5
C4 grass	0.17	0.082	6	43.5
Crops	0.17	0.156	10	39.8

Table 1 | Default and Revised Parameterizations for Sesquiterpene Emissions in MEGAN.
